# Computational scoring and experimental evaluation of enzymes generated by neural networks

**DOI:** 10.1038/s41587-024-02214-2

**Published:** 2024-04-23

**Authors:** Sean R. Johnson, Xiaozhi Fu, Sandra Viknander, Clara Goldin, Sarah Monaco, Aleksej Zelezniak, Kevin K. Yang

**Affiliations:** 1 New England Biolabs, Ipswich, MA USA; 2 Department of Life Sciences, Chalmers University of Technology, Gothenburg, Sweden; 3 Institute of Biotechnology, Life Sciences Centre, Vilnius University, Vilnius, Lithuania; 4 Randall Centre for Cell & Molecular Biophysics, King’s College London, Guy’s Campus, London, UK; 5 Microsoft Research, Cambridge, MA USA

**Keywords:** Machine learning, Protein design, Protein sequence analyses

## Abstract

In recent years, generative protein sequence models have been developed to sample novel sequences. However, predicting whether generated proteins will fold and function remains challenging. We evaluate a set of 20 diverse computational metrics to assess the quality of enzyme sequences produced by three contrasting generative models: ancestral sequence reconstruction, a generative adversarial network and a protein language model. Focusing on two enzyme families, we expressed and purified over 500 natural and generated sequences with 70–90% identity to the most similar natural sequences to benchmark computational metrics for predicting in vitro enzyme activity. Over three rounds of experiments, we developed a computational filter that improved the rate of experimental success by 50–150%. The proposed metrics and models will drive protein engineering research by serving as a benchmark for generative protein sequence models and helping to select active variants for experimental testing.

## Abstract (web summary)

Metrics that predict the success of folding and the activity of designed protein sequences are developed and experimentally validated.

## Main

Nature provides a wealth of proteins that can be used as biocatalysts to produce valuable products ranging from commodity chemicals to lifesaving pharmaceuticals^1^. Advances in DNA synthesis and recombinant DNA techniques have made it possible to clone genes that encode proteins into industrial organisms such as *Escherichia* *coli*^2^. Therefore, the use of recombinant proteins for industrial and therapeutic purposes has been highly successful^3,4^; however, the requirements associated with human applications are often not satisfied by natural proteins and engineering is needed to adapt them to human needs^5^.

A conventional method for moving beyond natural sequence and function space is to use directed evolution by starting from a natural protein and iteratively screening mutations until the protein acquires the desired properties^5,6^. Many mutations result in nonfunctional proteins^7,8^ and up to 70% of random single-amino acid substitutions result in decreased activity^9–12^. Computational models enable the generation of new and diverse sequences from a protein family, thereby uncovering previously untapped functional sequence diversity and reducing the number of nonfunctional sequences that need to be tested^13^. Typically, these generative models are trained either on large collections of protein sequences, for example the entire UniProt database of millions of sequences^14–16^, or on a set of proteins from a specific family^17,18^, with the goal of learning the training distribution to sample novel sequences with desired properties. The underlying assumption of these models is that natural proteins are under evolutionary pressure to be functional; therefore, novel sequences drawn from the same distribution will also be functional^19^. Many generative protein models have been proposed, including models based on deep neural networks, such as generative adversarial networks (GANs)^17^, variational autoencoders (VAEs)^18,20^, language models^15,16,21–24^ and other neural networks^25,26^, as well as statistical methods such as ancestral sequence reconstruction (ASR)^27,28^ and direct coupling analysis (DCA)^29–31^. However, comparing the ability of these methods to generate functional proteins remains a challenge because of limited experimental work evaluating model performance; likewise, there is no experimental validation supporting common computational metrics.

Typically, protein generative models are evaluated by comparing the distribution of generated sequences to natural controls using alignment-derived scores, for example, identity to the closest natural sequence^15,17^. The few reported results from biological assays^15,20,23,29,32,33^ used different experimental systems, making comparisons difficult because many factors can contribute to poor expression and activity (Supplementary Table 1), ranging from mutations disrupting protein folding and stability^34^ to codon usage hindering expression^35,36^. Thus, computational metrics for predicting the activity of generated sequences should account for as many factors as possible. For example, alignment-based metrics such as sequence identity or BLOSUM62 scores^37^ rely on homology to natural sequences and are good at detecting general sequence properties. However, they do not account for epistatic interactions and give equal weight to all positions^38^. In contrast, alignment-free methods do not require homology searches, are fast to compute and can potentially identify all sequence defects based on the likelihoods computed by protein language models^39^. Protein language models are sensitive to pathogenic missense mutations^40^, predict evolutionary velocity^41^ and capture viral immune-escape mutations^42^. Structure-supported metrics, including Rosetta-based scores^43^, AlphaFold2 (ref. ^44^) residue confidence scores and likelihoods computed by neural network inverse folding models^45–47^, use atomic coordinates to capture protein function; however, they can be expensive to use, especially when evaluating thousands of sequences. Although it is important to rationally choose metrics for computationally evaluating sequences, it is crucial to experimentally validate the ability of the metrics to predict function.

**Fig. 1:**
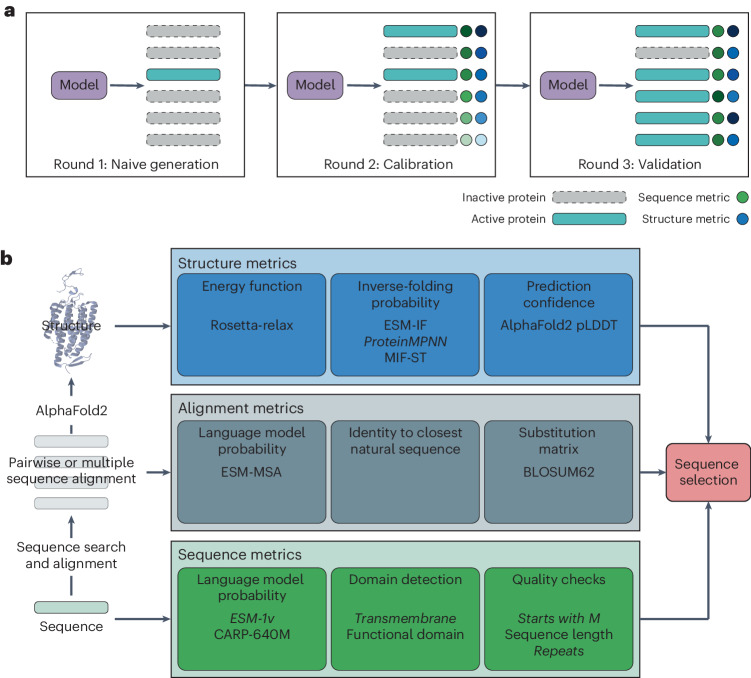
Study design. **a**, COMPSS was developed and tested over three rounds of experiments: (1) naive generation; (2) calibration; and (3) validation. **b**, COMPSS selects sequences using metrics calculated from single sequences, alignments and predicted structures. Metrics in italics were used in the final COMPSS filter.

In this study, we focused on assessing computational metrics to predict the functionality of computer-generated protein sequences. We experimentally evaluated in silico metrics for the ability to predict in vitro enzyme activity using sequences produced by three generative models trained on two enzyme families. Over three rounds of experiments (Fig. 1a) we developed and experimentally validated composite metrics for protein sequence selection (COMPSS), a framework that allows the selection of up to 100% of phylogenetically diverse functional sequences. COMPSS is generalizable to any protein family, and we provide examples as Google Colab notebooks. Our study demonstrates a composite computational metric for evaluating generated sequences that predicts experimental success. In addition to selecting active sequences for experimental validation, the proposed metrics are a first step toward establishing a standard for evaluating the performance of current and future protein generative models and will hopefully be a catalyst for driving progress in protein engineering.

## Results

In this work, a protein is considered experimentally successful if it can be expressed and folded in *E.* *coli* and has activity above background in an in vitro assay (Methods). We tested three protein generative models: (1) the transformer-based multiple-sequence alignment (MSA) language model ESM-MSA^48^; (2) a convolutional neural network with attention trained as a GAN (ProteinGAN)^17^; and (3) a phylogeny-based statistical model for ASR^28^. Although ESM-MSA is not trained as a generative model, it can be used to generate new sequences via iterative masking and sampling^49,50^. ASR is also not a truly generative model as it is constrained within a phylogeny to traverse backward in evolution without the ability to navigate sequence space in a new direction. However, it has successfully resurrected ancient sequences^51^ and increased enzyme thermotolerance^52^. To combine the strengths of the different methodologies, we considered alignment-based, alignment-free and structure-based metrics (Fig. 1b).

We experimentally evaluated the metrics on two enzyme families, malate dehydrogenase (MDH) and copper superoxide dismutase (CuSOD). Both MDH and CuSOD have substantial sequence diversity, have numerous members in the Protein Data Bank (PDB) and are physiologically significant^53,54^. They are also relatively small (300–350 residues for MDH and 150–250 residues for CuSOD) but are complex proteins active as multimers, and their activity can be assayed by spectrophotometric readout.

### Round 1: Naive generation results in mostly inactive sequences

For round 1, we constructed training sets to train the generative models of CuSOD and MDH. We collected 6,003 CuSOD and 4,765 MDH sequences from UniProt (Supplementary Table 2) with the Pfam domains for each protein family. CuSOD sequences had only a single Sod_Cu domain, whereas MDH sequences had an Ldh_1_N domain followed by an Ldh_1_C domain and no other Pfam domains. Nontypical domain architectures occurred in 6.3% and 1.7% of sequences in CuSOD and MDH, respectively. Sequences were truncated around the annotated domains to remove possible signal peptides, transmembrane domains and extraneous unannotated domains. Signal peptides are N-terminal leader sequences that facilitate secretion and are present in many proteins^55^. Signal peptides are frequently cleaved after secretion and are not present in the mature protein. In heterologous expression systems, signal peptides may not efficiently direct secretion or be cleaved, thereby interfering with protein expression^56^. Proteins with transmembrane domains are difficult to express and purify in heterologous systems^57^. We generated >30,000 sequences from the ASR, ProteinGAN and ESM-MSA models (Supplementary Table 3) and selected 144 sequences for experimental validation: 18 for each model and a set of natural test sequences. All generated and natural test sequences were selected to have 70−80% identity to the closest natural training sequence (Supplementary Table 4).

Of all experimentally tested sequences, including natural sequences, 19% were active (Extended Data Table 1 and Supplementary Figs. 1–6). None of the CuSOD ESM-MSA or test sequences and only two of the CuSOD GAN sequences were active. None of the MDH GAN or ESM-MSA sequences were active, but six of the MDH test sequences were active. In contrast, ASR generated 9 of 18 and 10 of 18 active enzymes for CuSOD and MDH, respectively.

We investigated the potential reasons for poor performance. We observed that natural test sequences with predicted signal peptides or transmembrane domains in the pretruncation sequences (Methods) were significantly overrepresented in the nonactive set (one-tailed Fisher test, *P* = 0.046). For CuSOD, a literature search^58,59^ combined with examination of the assayed sequences and the available CuSOD crystal structure (PDB: 4B3E)^60^ showed that CuSOD is active as a homodimer (or sometimes a tetramer) and that the truncations we made to the natural sequences often removed residues at the dimer interface, likely interfering with expression and activity. Thus, we made equivalent truncations to our positive-control enzymes, human SOD1 (ref. ^61^) (hSOD, GenBank: NP_000445.1), *Potentilla* *atrosanguinea* CuSOD^62^ (paSOD, GenBank: AFN42318.1) and *E.* *coli* SOD^63^ (E.SOD, GenBank: NP_416173.1), and confirmed loss of activity for hSOD and paSOD (Supplementary Figs. 7 and 8). Overtruncation also affected ASR sequences, yet many were still active, possibly due to the widely reported stabilizing effect of ASR^27,64,65^.

To further test the hypothesis that overtruncation led to a lack of activity in the round 1 natural CuSOD test sequences, we assayed an additional 14 natural CuSOD proteins and 2 proteins from the evolutionarily distinct FeSOD family (pretest group). In nature, eukaryotic CuSOD proteins are typically cytosolic and lack a signal peptide, whereas bacterial CuSOD proteins are typically secreted via a signal peptide^58^. CuSOD sequences were selected on the basis of kingdom (eukaryotic, viral or bacterial), and the presence of a signal peptide was predicted using Phobius^66^. Sequences with predicted signal peptides were truncated at the predicted cleavage site. Both of the chosen bacterial FeSOD proteins lacked a predicted signal peptide, as does *E.* *coli* FeSOD^63^ (SodB, GenBank: NP_416173.1, positive control). Activity was noted in 8 of the 14 CuSOD sequences, including 3 of the 4 eukaryotic enzymes and a single viral enzyme, all of which lacked a predicted signal peptide and were expressed in their full-length form (Supplementary Figs. 9−11). Three of the seven signal peptide-clipped bacterial CuSOD enzymes also had activity. For MDH, overtruncation was less problematic and 6 of 17 natural test sequences were active (Extended Data Table 1 and Supplementary Figs. 1a, 2a and 3a).

### Round 2: Calibration data for COMPSS

Consolidating the lessons learned in round 1, we retrained the models and tested additional sequences to calibrate the computational metrics. Specifically, for the training set and natural test sequences, we used only full-length natural sequences, removing sequences with predicted transmembrane domains and signal peptides. We also increased the identity band, choosing generated sequences with 80−90% identity to the closest training sequence. For CuSOD, we selected only eukaryotic or viral proteins (Supplementary Table 2). For sequence generation, we used the same method as in round 1 for generating ASR and GAN sequences but modified the ESM-MSA sampling procedure to improve the quality of generated sequences (Supplementary Table 3). ESM-MSA sampling for round 1 used MSAs composed of randomly selected training sequences masked and sampled across the entire MSA. For round 2, only one training sequence from the MSA was masked and sampled at a time based on an MSA composed of the training sequences most similar to the resampled sequence. Sequences generated with the revised ESM-MSA sampling method had higher metric scores, including ESM-1v and identity to the closest training sequence (Supplementary Fig. 12d). We selected 18 sequences each from ASR, GAN and ESM-MSA. Only 13 natural test sequences were selected, because we had already screened 5 similar natural sequences in the remediation for round 1. Natural test sequences were selected using the same criteria as used for model-generated sequences. The number of expressed enzymes with activity above the background was substantially higher than that in round 1, with 66% of natural controls showing activity when expressed in *E.* *coli*, and at least 50% of generated sequences were active for every model−enzyme family combination except for GAN−MDH, where only 2 of 18 sequences were active (Extended Data Table 1 and Supplementary Figs. 13–16).

**Fig. 2:**
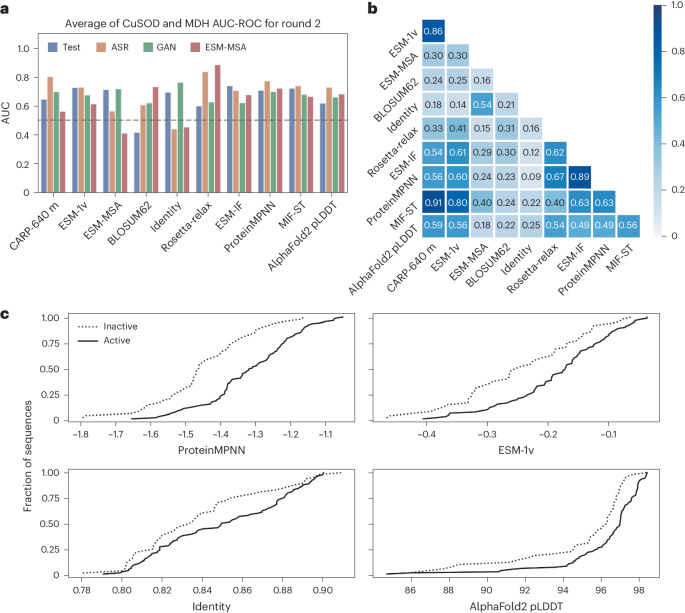
Computational metrics of sequences experimentally tested in round 2. **a**, AUC-ROC scores of activity versus metrics. The average of CuSOD and MDH. **b**, Spearman correlations between metrics. The average of CuSOD and MDH. **c**, Empirical cumulative distribution functions of selected metrics for active (solid lines) and inactive (dashed lines) sequences. Curves represent the pooled results of all generative models and both enzyme families.

To calibrate the metrics against enzymatic activity, we computed alignment-based (identity, BLOSUM62 (ref. ^37^), PFASUM15 (ref. ^67^), phmmer top 30 average score, ESM-MSA) and sequence-only alignment-free (CARP-640M^68^, ESM-v1 (ref. ^39^), net charge^23^, abs(net charge), charged fraction) metrics. We also predicted AlphaFold2 structures^44,69^ and used the predicted structures to calculate structure-based Rosetta energies^43^, solvent-accessible surface area (SASA)^23,70^, ProteinMPNN^45^, ESM-IF^46^ and MIF-ST^47^. The experimentally tested sequences were selected to span the entire range of scores for each metric (Supplementary Table 4). To identify metrics capable of detecting failure modes undetectable by an expert human, we manually excluded candidates with large insertions, deletions or long repeats and added an N-terminal methionine to seven generated sequences. Apart from the alignment-based ESM-MSA metric, none of the metrics strongly correlated with sequence identity, suggesting that our chosen metrics are orthogonal to sequence identity (Supplementary Fig. 17). In contrast, structure-based metrics were substantially correlated, with the highest correlations between inverse folding neural network scores (Fig. 2b and Supplementary Fig. 18).

Area under the curve receiver operating characteristic values (AUC-ROCs) between activity and each metric (Fig. 2a, Table 1 and Supplementary Figs. 18−20) indicate that the metrics are predictive of activity, but none stands out as superior to the others. Inverse folding metrics, on average, best predicted enzymatic activity, showing an AUC-ROC of 0.72 when combining all models and families. AlphaFold2 residue confidence pLDDT scores were significantly predictive for CuSOD (Fig. 2c; Wilcoxon rank sum *P* = 5 × 10^−4^), but not for MDH (Table 1). Sequence identity did not predict activity (Table 1, Fig. 2c and Supplementary Fig. 18).

**Table 1:** AUC-ROCs of each metric versus experimentally measured activity in round 2

Input	Metric type	Metric	CuSOD					MDH					Average
			Test	ASR	GAN	ESM-MSA	All	Test	ASR	GAN	ESM-MSA	All	All
Single sequence	Residue counting	Net charge	0.73	0.24	0.28	0.28	0.30	0.57	0.50	0.21	0.51	0.48	0.39
		Abs(net charge)	0.27	0.76	0.72	0.75	0.70	0.18	0.17	0.75	0.49	0.39	0.55
		Charged fraction	0.44	1.00	0.42	0.69	0.57	0.36	0.47	0.33	0.49	0.43	0.50
	Language model	CARP-640M	0.73	0.94	0.74	0.70	0.76	0.57	0.67	0.66	0.43	0.60	0.68
		**ESM-1v**	**0.85**	**0.88**	**0.69**	**0.75**	**0.76**	**0.61**	**0.58**	**0.66**	**0.48**	**0.60**	**0.68**
		ESM-1v mask6	0.83	0.94	0.71	0.76	0.78	0.55	0.53	0.50	0.48	0.53	0.66
		ESM-MSA	0.63	0.53	0.65	0.37	0.53	0.80	0.60	0.79	0.45	0.70	0.61
Sequence alignment	Substitution matrix	Avg(phmmer top 30)	0.60	0.41	0.60	0.79	0.68	0.61	0.40	0.71	0.60	0.50	0.59
		BLOSUM62	0.38	0.62	0.67	0.71	0.61	0.46	0.60	0.57	0.75	0.63	0.62
		PFAMSUM15	0.35	0.18	0.63	0.68	0.61	0.46	0.60	0.54	0.75	0.62	0.62
	Identity	Identity	0.59	0.35	0.74	0.65	0.62	0.80	0.53	0.79	0.26	0.60	0.61
Structure	Energy function	Rosetta-relax	0.65	0.94	0.67	0.89	0.78	0.55	0.73	0.59	0.88	0.75	0.76
	Inverse folding	ESM-IF	0.75	0.88	0.64	0.85	0.76	0.73	0.53	0.61	0.51	0.65	0.70
		**ProteinMPNN**	**0.78**	**0.88**	**0.65**	**0.90**	**0.80**	**0.64**	**0.67**	**0.75**	**0.55**	**0.70**	**0.75**
		MIF-ST	0.75	0.88	0.68	0.79	0.77	0.70	0.60	0.68	0.55	0.68	0.72
	Surface area	SASA	0.28	0.18	0.43	0.24	0.30	0.59	0.18	0.32	0.58	0.47	0.39
		Polar SASA	0.28	0.53	0.56	0.31	0.40	0.38	0.24	0.46	0.62	0.50	0.45
		Apolar SASA	0.30	0.12	0.35	0.23	0.27	0.71	0.27	0.25	0.56	0.47	0.37
		Percent polar SASA	0.40	0.88	0.75	0.49	0.60	0.29	0.42	0.52	0.57	0.52	0.56
	Prediction confidence	AlphaFold2 pLDDT	0.77	0.88	0.61	0.88	0.77	0.46	0.58	0.71	0.49	0.55	0.66

The ESM-1v and ProteinMPNN metrics shown in bold were selected to be part of the filter for round 3.

### Round 3: Validation: COMPSS enriches active protein generation

**Fig. 3:**
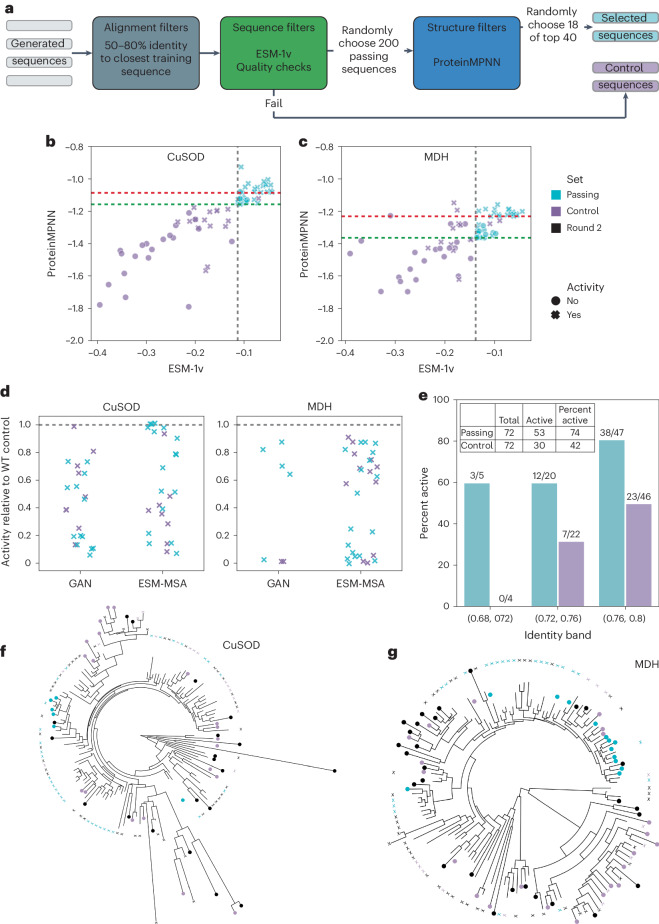
Round 3 selection and experimental results. **a**, Computational filter for selecting sequences to screen in round 3. **b**,**c**, CuSOD (**b**) and MDH (**c**) ESM-1v andProteinMPNN scores for round 3 selected passing (teal) and control (violet) sequences. The vertical dashed gray line indicates the top 10th percentile cutoff for ESM-1v score calculated from the test sequences. Horizontal dashed lines are the ProteinMPNN scores of the 40th-ranked sequences among the 200 selected sequences sequences for which the scores were calculated for each model−family combination, including ProteinGAN (lower, green) and ESM-MSA (upper, red). Control sequences to the right of the gray line are possible if they failed one of the quality checks. **d**, Comparison of the specific activities of active generated sequences versus wild-type (WT) controls (Methods). **e**, Active enzymes by identity band. **f**, Phylogenetic tree of CuSOD enzymes screened in round 2 (black) and round 3 (passing, teal; control, violet). An X indicates an active sequence and a filled circle indicates an inactive sequence. **g**, Phylogenetic tree of MDH sequences.

Next, we devised an in silico filter to virtually screen large numbers of generated sequences to identify probable active sequences with <80% identity to the closest natural sequence. Based on round 2 (Fig. 2, and Table 1), no single metric was sufficiently general against multiple failure modes (Supplementary Table 1); therefore, we tested a filter composed of a combination of the ESM-1v and ProteinMPNN metrics (Fig. 3a and Supplementary Fig. 21). This combination was attractive because ESM-1v is sequence based, ProteinMPNN considers structural information and neither metric is strongly correlated with sequence identity (Fig. 2a). Although most inverse folding or energy function-based metrics performed similarly (Table 1), ProteinMPNN was the most computationally efficient. Rosetta-relax performed best on MDH sequences but is more computationally expensive than other structure-based metrics. The fast-to-compute ESM-1v protein language sequence model showed the best performance for alignment-free metrics, with an average AUC-ROC of 0.68. Furthermore, the two metrics were only moderately correlated, with Spearman’s *ρ* = 0.60 (Fig. 2a), suggesting that they capture distinct features.

To select a threshold for ESM-1v scores, we analyzed the results for the natural test sequences from round 2. We found that the highest enrichment of active sequences occurred at approximately the 20th percentile of the ESM-1v scores (the top 38% and 17% for CuSOD and MDH, respectively) (Supplementary Fig. 22). For prospective validation of our sequence prioritization strategy, in round 3, we used the 10th percentile of the natural sequences, making the threshold more stringent because, in practice, the score should be derived from untested natural sequences. To validate our strategy, we focused on the GAN and ESM-MSA models because ASR sequences performed consistently well in rounds 1 and 2 and in the literature. The filter begins with automated quality checks for sequences starting with methionine and lacking long repeats and transmembrane domains, intended to approximate human intuition. Next, we randomly selected 200 ESM-1v threshold-passing sequences with 50−80% identity to natural sequences for each model and enzyme family, predicted their structure using AlphaFold2 and randomly selected 18 of the top 40 sequences based on ProteinMPNN scores for each model and enzyme family combination. For each sequence selected for experimental validation, a negative control was randomly chosen from the sequences failing the ESM-1v filter with an identity to the closest training sequence within 1% of that for the passing sequence. The stringency of the ESM-1v filter led to phylogenetic bias, particularly for MDH; nevertheless, the set of screened enzymes covered approximately the same space in round 3 as in round 2 (Fig. 3f,g and Supplementary Figs. 23 and 24).

A total of 144 selected and control sequences were expressed in *E.* *coli*, purified and assayed for activity (Supplementary Figs. 25–30). The selected enzymes had an identity to the closest natural sequence of >69%. Most of the selected sequences showed in vitro activity, including 94% (17 of 18) and 100% of ESM-MSA CuSOD and MDH enzymes, respectively (Fig. 3b–e and Extended Data Table 1). Furthermore, when combining sequences from both models and enzyme families, 74% were active, which is a 77% higher success rate (two-tailed Fisher test, *P* = 0.00018) than for the sequence-filter-failing control sequences (Fig. 3e and Supplementary Fig. 31). Furthermore, 83% (44 of 53) of active generated sequences selected by COMPSS had activity levels within an order of magnitude of those of the wild-type controls (Fig. 3d), suggesting that COMPSS enriches sequences of sufficient activity and diversity to be possible starting points for engineering with directed evolution.

We also used ProGen^15^, a 1.2-billion-parameter protein language model, to generate lysozyme sequences from the glucosaminidase and transglycosylase families. We selected 18 passing and 18 identity-matched controls from each family, as well as 12 previously reported active lysozymes, four natural and eight generated by ProGen. In our study, 14 of 84 sequences expressed and could be purified from *E.* *coli* (Supplementary Fig. 32), but only the previously reported L056 and L070, generated by ProGen from the phage lysozyme (PF00959) family, showed activity (Supplementary Table 6). The ProGen study used multiple language models, including TAPE-BERT discriminators fine-tuned on lysozymes, to select sequences, suggesting that generating and selecting active enzymes from ProGen requires fine-tuning both the generative and discriminative models for each family.

**Fig. 4:**
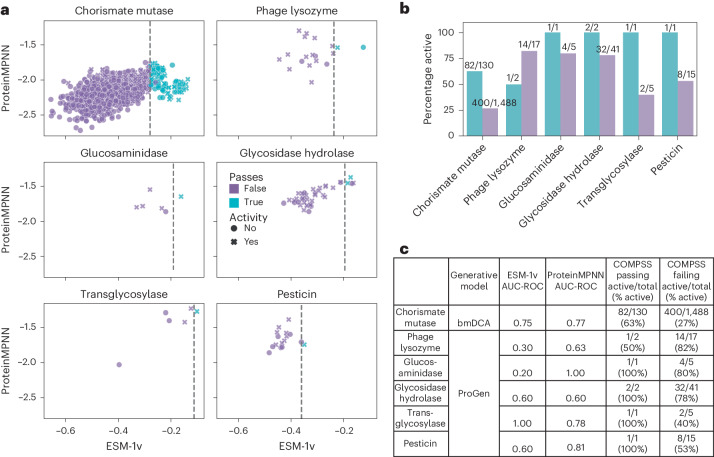
Validation of COMPSS against enzymes generated from six previously unseen protein families and two new models. **a**, ESM-1v and ProteinMPNN scores of generated enzymes from the six families. The vertical dashed gray line indicates the top 10th percentile cutoff for ESM-1v score calculated from natural sequences from the same families. **b**, Proportion of active enzymes stratified by family and whether they passed the COMPSS sequence filter. **c**, COMPSS performance of the six families from publicly available data.

To further validate COMPSS, we tested it against previously published datasets of experimentally characterized sequences from six additional families generated by models with different architectures from those trained in this work (Supplementary Table 5), including enzymes from five evolutionarily distinct lysozyme families generated by ProGen^15^ and chorismate mutases generated by bmDCA^29^. When applying COMPSS to these sequences, we omitted the identity and ‘starts with M’ filters as they would eliminate most sequences in these datasets. For five of the six families, there was a higher fraction of functional enzymes among those passing the COMPSS sequence filter than among those failing the filter, and ProteinMPNN AUC-ROCs ranged from 0.6 to 1.0 (Fig. 4).

The critical importance of training data curation is evident from the improved success rates between round 1, round 2 and control round 3 sequences. Furthermore, none of the 42 natural and 61 synthetic chorismate mutases with predicted signal peptides were active. Simple sequence quality checks also contributed to the success rate. Applying the round 3 sequence quality checks to round 2 sequences showed that 13 of 24 (54%) check-failing sequences were active, compared with 78 of 115 (68%) check-passing sequences. In round 3, 10 of 21 (48%) check-failing negative-control sequences were active, compared with 20 of 51 (39%) check-passing negative-control sequences, and 53 of 72 (74%) of sequences selected by the combined COMPSS filter, including quality checks, ESM-1v scores and ProteinMPNN scores.

To deconvolve the relative contributions of ESM-1v and ProteinMPNN to the success of the round 3 selections, we considered sequences generated and assayed in rounds 2 and 3 (which used the same training sets and generative models), as well as the chorismate mutase and lysozyme literature datasets. We divided the CuSOD, MDH and chorismate mutase datasets into quadrants based on median ProteinMPNN and ESM-1v scores and calculated the metric AUC-ROCs, Spearman correlations and percentage of active enzymes for the whole datasets as well as for each quadrant (Supplementary Fig. 33). The ProteinMPNN to ESM-1v Spearman correlation was higher in the whole dataset than in any individual quadrant. Furthermore, the lower-left quadrant (lowest scores on both metrics) had the lowest success rate. For chorismate mutase and CuSOD, the upper-right quadrant (highest scores on both metrics) had the highest success rates, whereas for MDH, the upper-left and upper-right quadrants (high ProteinMPNN scores) had similar success rates. Thus, it is clear that sequences with low ESM-1v scores are likely to also have low ProteinMPNN scores and be inactive, confirming that only computing ProteinMPNN scores for sequences with high ESM-1v scores and selecting candidates from the top right quadrant efficiently improves success rates while limiting the number of expensive structure predictions.

The tested lysozyme sequences all passed a stringent filter based on TAPE-BERT and ProGen: there were no negative controls^15^. Thus, we treated them as though they had already passed through a language model filter and were comparable to the two right-hand quadrants (ESM-1v score above the median) of the other datasets and compared the statistics of sequences with ProteinMPNN scores above and below the median (Supplementary Fig. 33). We observed trends similar to those in the half with high ESM-1v scores of the other datasets. Combining the five lysozyme families, sequences with a ProteinMPNN score above the median had a 38% higher success rate than those below the median (39 of 46 versus 27 of 44). Therefore, Madani et al.^15^ may have improved their success rate by including a filter based on an inverse folding model. ProteinMPNN provides orthogonal information, increasing the success rate over language model-based selection alone.

We also used the published datasets to assess the effectiveness of an ESM-1v- and ProteinMPNN-based filter on sequences having less than 70% identity to the closest training sequence. Dividing the sequences with less than 70% identity into quadrants, we observed the same trends as in the full dataset (Supplementary Fig. 34), with AUC-ROCs of 0.91 and 0.92 for ProteinMPNN and ESM-1v for the chorismate mutase data, respectively, and 0.67, 0.67 and 0.67 for ProteinMPNN on the three lysozyme datasets with more than one sequence in the bin with less than 70% identity.

## Discussion

Discovering new enzymes is difficult because it requires an understanding of the molecular mechanisms, expression and folding while operating within biological and physical constraints. Generative protein sequence models mimic these constraints by learning to sample from natural sequence distributions. Deep neural networks have led to advances in generative protein design^71^, enabling the design of de novo proteins with specified folds^45,72^. Because of the emergent complexity underlying catalysis (Supplementary Table 1), predicting which enzyme sequences will express and fold in soluble and active forms remains challenging, limiting the discovery of novel enzymes. To facilitate enzyme discovery from non-natural sequence spaces, we experimentally evaluated a diverse set of in silico metrics to determine their efficacy in predicting sequence activity. We selected a subset of the metrics to form COMPSS. We validated the filter’s effectiveness by prospectively testing synthetic proteins, which resulted in up to a twofold increase in the number of active protein sequences (Extended Data Table 1). Similar results were obtained by independently validating COMPSS on previously published datasets^15,29^ of six enzyme families generated by models not trained in this study (Fig. 4).

Our experimentally validated end-to-end framework for generating and selecting new active enzyme variants (Tables 1 and 2) consists of three steps. The first step is curating sequences to obtain a high-quality training dataset. Machine learning is a data-centric practice as much as it is algorithmic; therefore, dataset curation was crucial. Neither ESM-MSA nor the local and much smaller ProteinGAN model performed well in the naive round 1 setting (Extended Data Table 1). In round 2, we observed that removing sequences containing transmembrane domains and signal peptides enriched the dataset for soluble proteins and allowed both models to generate active enzymes at rates above 60%. In round 2, we also selected sequences with a broad range of scores on the metrics and observed which metrics predicted activity (Fig. 2). For the round 3 validation measurements, we combined sequence-based quality checks, the sequence-based ESM-1v metric and the structure-based ProteinMPNN metric to select generated sequences, resulting in enrichment of active sequences (Fig. 3a and Supplementary Fig. 21). While AlphaFold2 (ref. ^44^) accurately predicts protein structures from MSAs^73^, the residue confidence pLDDT score does not consistently predict enzyme activity within sets of homologous synthetic sequences (Table 1 and Supplementary Fig. 18). AlphaFold2 predicts high-confidence structures even for inactive sequences (Fig. 2c and Supplementary Fig. 17). Likewise, for sequences with 70–90% identity to natural sequences, neither identity nor sequence similarity captures functional differences (Table 1 and Fig. 2a,c). In contrast, likelihoods from protein language models and inverse folding models moderately predicted in vitro enzyme activity and were weakly correlated with each other (Table 1 and Fig. 2a). Therefore, we used one language model (ESM-1v) and one inverse folding model (ProteinMPNN) in COMPSS.

**Table 2:** Overview of the steps and considerations in a typical workflow for generating new active enzyme variants

Step	Description	Examples and considerations
Curate training data	Gather a list of natural sequences likely to have the target activity and express in the target system.	In addition to UniProt and/or NCBI nr, search expanded databases, such as Mgnify for prokaryotic enzymes or NCBI TSA for eukaryotic enzymes.
		Pay attention to the domain content. Unusual domain content indicates neofunctionalization. In some cases, the domain with the activity of interest will retain its function in the absence of the other domains; therefore, it may be safe to remove extraneous domains.
		Pay attention to the presence of localization tags and transmembrane domains. In many cases, these interfere with expression. In some cases, they can be removed without impacting enzyme function.
		Filter out unusually short or long sequences, or sequences with other indications that they may be pseudogenes, fragments or derived from poor gene calling.
		Use hmm-profile or structure searches in addition to sequence searches to find a broader diversity of training sequences.
		Use a clustering algorithm, such as CD-HIT, to reduce the overrepresentation of enzymes from highly sequenced phyla.
Generate new sequences	Use generative models to generate additional members of the enzyme family. Most of these generative models rely on training or fine-tuning of natural sequences curated in the first step of the workflow.	ASR
		Generative adversarial networks (ProteinGAN)
		Language models (such as ProtGPT2, ProGen or ESM-MSA
		VAE
		DCA-based methods
		Inverse folding models (ProteinMPNN, ESM-IF)
Select sequences	Select a subset of natural and generated sequences for experimental evaluation. In campaigns where all sequences are natural or ancestral reconstructions, random selection of candidates may be effective, particularly if care is taken in training data curation. For generative models that produce a lower proportion of active sequences, additional filtering may be required.	Randomly select candidate sequences.
		Select sequences with high similarity to the best candidates from previous screening rounds or the literature (phylogeny-based selection).
		Select sequences with mutations known to be associated with the target phenotype.
		The same curation criteria for natural sequences are also applicable to generated sequences.
		Additional criteria may be used to address failure modes common to the generative models used. For example, models may tend to produce overly short or repetitive sequences.
		Sequences can be scored and ranked based on various metrics. Reasonable scores for these metrics can be estimated from natural sequences. Alternatively, candidates can be selected from the highest-scoring sequences.
		In this study, we settled on a filter composed of six criteria.

Our study does not benchmark generative models against each other but instead evaluates metrics to identify those widely applicable across models and enzyme families. Nevertheless, we found that ASR outperforms neural network models in naive generation, indicating room for improvement in protein deep generative models. We observed a wide range of AUC-ROC scores for different combinations of the generative model, enzyme family and metric. Different models and enzyme families may have different failure modes captured by different metrics. Some metrics use underlying models similar to the generative models, which may lead to overfitting. Despite evaluating over 2,200 enzyme variants from eight families, given the sheer size of the protein sequence space, a more extensive dataset could help tease apart the complex interplay between generative models, metrics and protein families and explain that interplay in biologically meaningful terms.

The core idea of COMPSS is to select sequences by prefiltering with fast, biologically motivated quality checks and protein language model scores, followed by a slower step of structure prediction and inverse folding model scoring. Many variations on this core idea are possible, and we do not recommend blindly applying COMPSS to new protein families without considering their biological complexities. In addition to adjusting the ESM-1v score cutoff using natural sequences, biologically motivated quality filters should be chosen on a per-family basis. For example, long repeats or transmembrane domains are required for function in some protein families. The ‘starts with M’ filter was a good marker of full-length sequences for our dataset because all training sequences started with methionine. In cases where the training sequences have N-terminal truncations, a minimum length filter would similarly eliminate fragments.

We showed that, by carefully curating training data for sequence generation and prioritizing sequences for experimental testing using a multipart filter, as high a proportion as 100% of enzymes with in vitro activity can be achieved, with sequence identities between 70% and 80% to the closest naturally occurring enzymes. We provide Google Colab notebooks for generating new sequences using ESM-MSA and calculating metrics for any user-supplied sequences or structures. Our dataset of more than 500 experimentally tested enzymes and metrics can serve as reference benchmarks for predicting the function of the generated sequences. The presented end-to-end workflow provides a powerful and flexible framework for generating diverse libraries of active enzymes, enabling deeper explorations of the functional sequence space.

## Methods

See the Supplementary Methods for additional details throughout.

### Data curation

#### Round 1 CuSOD

UniProt^74^ sequences containing exactly one Sod_Cu Pfam^75^ domain were downloaded. Hmmsearch (Hmmer, http://hmmer.org/; ref. ^76^) identified the Sod_Cu domain envelopes. Sequences were truncated to remove extraneous sequences beyond the bounds of the Sod_Cu match. Additional quality filtering was performed. Sequence duplicates were removed using CD-HIT^77^ at an identity threshold of 80%, and 80% and 20% were randomly sorted into a ‘training’ and a ‘test’ set, respectively. A training MSA was generated by an iterative process using MUSCLE (v3.8)^78^.

#### Round 1 MDH

All UniProt sequences containing an Ldh_1_N Pfam domain followed by an Ldh_1_C domain were downloaded. LDH and MDH enzymes, based on enzyme commission number^79^, 1.1.1.27 for LDH and 1.1.1.37 for MDH, were downloaded from SwissProt. MUSCLE and hmmbuild were used to build profile hidden Markov models of both sets. Hmmsearch was used to score each UniProt Ldh_1_N/Ldh_1_C sequence against the MDH and LDH profiles and sequences that had a stronger match to the MDH profile were retained. Additional processing was performed exactly as with the round 1 CuSOD data curation.

#### Quantification of domain architectures

See the Supplementary Methods.

#### Round 2 CuSOD pretest

UniProt CuSOD proteins were obtained as described above (round 1 CuSOD). The kingdom of origin for each sequence was obtained from the UniProt annotation. Transmembrane domains and signal peptides were predicted using Phobius^66^. Sequences with transmembrane domains were discarded. Signal peptides were removed from sequences predicted to contain them. A set of 14 representative CuSOD and 2 FeSOD proteins were manually selected for experimental screening, including eukaryotic, viral and bacterial proteins predicted to not contain signal peptides, and bacterial proteins with predicted signal peptides removed.

#### Rounds 2 and 3 CuSOD

All eukaryotic transcriptomes available from the NCBI Transcriptome Shotgun Assembly (TSA) sequence database^80^ were downloaded. Transdecoder (https://github.com/TransDecoder/TransDecoder) was used to extract the protein sequences from transcriptomes. Hmmsearch^76^ was used to identify proteins with exactly one Sod_Cu domain. This set of proteins was combined with the list of eukaryotic and viral CuSOD proteins from UniProt. Additional quality filtering was performed. Sequences that were more than 85% identical (based on usearch^81^ search_global) to a sequence screened in a previous round were discarded. The remaining sequences were deduplicated at 90% using CD-HIT and then split 90% and 10% into training and test groups, respectively. A training MSA was generated.

#### Rounds 2 and 3 MDH

Hmmsearch^76^ was used to search Mgnify^82^ for sequences containing exactly one Ldh_1_C and one Ldh_1_N domain. The list of Mgnify proteins was added to the list of UniProt (curation described above). Additional quality filtering was performed. Sequences were deduplicated at 90% using CD-HIT. Sequences with identity greater than 85%, based on usearch search_global, to a sequence experimentally screened in round 1 were discarded. The remaining sequences were split into training (90%) and test (10%) sets. A training MSA was generated.

#### Phylogenetic trees

Trees were constructed using FastTree from MSAs generated by MAFFT^83^. Trees were rooted and the midpoint was rendered using ETE3 (ref. ^84^).

#### Chorismate mutase and lysozymes

See the Supplementary Methods.

### Generative models

#### ESM-MSA-1b sampling

Sequences were generated by iterative masking and sampling using the ESM-MSA-1b model^48^. ESM-MSA-1b is a neural network model trained to fill in the wild-type amino acids in masked positions of a protein MSA. The model can be used to generate new sequences by running MSA masking and prediction iteratively, each time replacing the wild-type amino acids at the masked positions with an amino acid drawn from the probability distribution returned by the model. The use of masked language models to generate new sequences was first proposed by Wang and Cho^50^, and the strategy has been applied to protein sequences in at least three prior works^22,49,85^.

See the Supplementary Methods for more detail on the parameters used.

#### ProteinGAN

Generative adversarial models were trained using the training sets for CuSOD and MDH. Then, for each family, sequences were generated by sampling vectors from the latent space using a truncated normal distribution. For rounds 1 and 2, 10,048 sequences were generated for each family. For round 3, 560,016 and 160,064 sequences were generated for CuSOD and MDH, respectively.

#### Ancestral sequence reconstruction

Maximum-likelihood trees were generated from the training set reference MSAs using FastTree^86^. Ancestral sequence reconstructions were generated from the trees using the joint reconstruction function of the GRASP^28^ command line tool. Metrics were calculated, and candidates were selected from the entire set of reconstructed sequences.

#### ProGen

See the Supplementary Methods.

### Computational metrics

#### AlphaFold2

AlphaFold2 (ref. ^44^) was used to predict the structures of test sequences and all generated sequences that passed the first filtering step.

#### Phobius

The jphobius^66^ (https://phobius.sbc.su.se/data.html) executable was used to predict the presence of signal peptides or transmembrane domains.

#### ESM-1v and CARP-640M

Scores calculated from the ESM-1v^39^ and CARP-640M^68^ models were the average of the log probabilities of the amino acid in each position. Without masking, this calculation can be done with a single forward pass over each sequence. With partial masking, it can be done in a number of passes equal to one per masked_fraction.

#### ESM-MSA

Scores from the ESM-MSA-1b^48^ model were calculated in a manner similar to that for ESM-1v scores, using the average log probability across the whole sequence. The metric was calculated using phmmer^76^ to find the 31 closest training sequences to each query, align the 32 sequences with MAFFT and calculate the average log probabilities from six passes with a masking interval of six.

#### ProteinMPNN, ESM-IF and MIF-ST

The proteinMPNN^45^ and ESM-IF^46^ scores are the average log likelihood of the query residues using the AlphaFold2-predicted structure. The MIF-ST^47^ score was calculated using the extract_mif.py script from the protein sequence models repository (https://github.com/microsoft/protein-sequence-models).

#### Rosetta-relax

The Rosetta (v2020.08.61146)^43^ relax program was used to relax the AlphaFold2 structures.

#### Distance to the closest training sequence

The most similar training sequence was found using ggsearch36 from the FASTA package^87^, the BLOSUM62 scoring matrix and a gap open penalty of 10 and gap extend penalty of 2. The Hamming distance was then calculated from the gapped alignment between the query and the top hit sequences. Identity was calculated as 1 − Hamming_distance.

#### BLOSUM62 and PFASUM15 mutant position mean

The closest training sequence was found using ggsearch36 as described above. From the alignment to the closest training sequence, the mean BLOSUM62 score^37^ across all mismatched positions was calculated, ignoring positions where either the query or the reference had a gap. We also calculated the alignments and scores using an alternative matrix, the PFASUM15 matrix^67^.

#### Longest repeat

Scores were calculated for the longest single-amino acid repeat and the longest 2-mer, 3-mer and 4-mer repeat in each sequence. The scores were calculated as −1 ⨯ the number of repeat units. Therefore, the sequence AAAAAA would have a single-amino acid repeat score of −6, a 2-mer score of −3, a 3-mer score of −2 and a 4-mer score of −1. The sequence LALALALA would have a 1-mer score of −1, a 2-mer score of −4, a 3-mer score of −1 and a 4-mer score of −2.

#### SASA

SASA, polar SASA and apolar SASA were calculated from the AlphaFold2-predicted structures using the freesasa package (https://freesasa.github.io/). The percentage of polar SASA was calculated using the formula 100 ⨯ polar SASA/SASA.

#### Net charge, Abs(net charge) and charged fraction

Charges were calculated by summing the numbers of glutamate and aspartate residues and lysine and arginine residues for negative and positive charges, respectively.

#### Avg(phmmer top 30)

The phmmer top 30 average score was calculated by running a phmmer search of the experimentally tested sequences against the training sequences and averaging the scores of the top 30 hits.

### Selection of sequences for in vitro assays

#### Round 1

The selected sequences had 70% and 80% identity to the closest training sequence and diverse scores on the ESM-1v metric.

#### Round 2 pretest

CuSOD sequences were selected on the basis of the kingdom of origin (eukaryotic, viral or bacterial) and the presence of Phobius-predicted signal peptides. Sequences with predicted signal peptides were truncated at the predicted signal peptide cleavage site. Two bacterial FeSOD proteins, both lacking a predicted signal peptide, and the previously characterized^63^ *E.* *coli* FeSOD (as a positive control) were also assayed.

#### Round 2

The selected sequences had between 80% and 90% identity to the closest training set sequence and diverse scores on the ESM-1v and ESM-MSA metrics. Sequences were also filtered by manual inspection to remove those with large insertions or deletions compared to the closest reference sequences or long repeats, and a methionine was added to the start of a few of the sequences.

#### Round 3

Sequences were selected on the basis of a series of filters. The first filter removed sequences having (1) less than 50% or greater than 80% identity to the closest training sequence; (2) an ESM-1v score below the top 10th percentile threshold compared to the test sequences; (3) no starting methionine; (4) a predicted transmembrane domain; and (5) a single-amino acid repeat longer than three amino acids or an amino acid pair repeat longer than four amino acids, as repeats were more common in ESM-MSA-generated sequences than in natural sequences (Supplementary Fig. 35). For each enzyme family, 200 ESM-MSA-generated sequences and 200 GAN-generated sequences were randomly selected from the sequences that passed the first filter, and their structures were predicted with AlphaFold2. ProteinMPNN scores were calculated for each structure, and the 40 sequences with the highest scores from each model−enzyme combination were retained. Of the top 40 sequences, 18 were randomly selected for expression and functional characterization. For each passing sequence that was selected for functional characterization, a corresponding control sequence was selected from the list of sequences that failed the sequence filter. Control sequences were identical to the closest training sequence within 1% of the passing sequence.

#### Newly generated ProGen lysozyme sequences

See the Supplementary Methods.

### Experimental assays

#### Bacterial strains, plasmids and growth conditions

*E.* *coli* BL21(DE3) was used as the host strain for MDH and SOD expression in this study. Cells were grown on LB medium at 37 °C and supplemented with 100 μg ml^−1^ ampicillin (cat. no.171254, Merck).

Sequences were optimized based on *E.* *coli*-preferred codons using the Twist Bioscience web interface (www.twistbioscience.com). A 30-bp sequence (TTTGTTTAACTTTAAGAAGGAGATATACAT) composed of ribosomal binding site sequences and a spacer were added at the 5′ terminus of all genes. Genes were ordered from Twist Bioscience as clones in pET-21(+) between the EcoRI and NotI sites.

The pET21b plasmid harboring the MDH4 gene from a previous study^17^ was used as a positive control for MDH enzymes. Human SOD1 (ref. ^61^) (hSOD, GenBank: NP_000445.1), *Potentilla* *atrosanguinea* CuSOD^62^ (paSOD, GenBank: AFN42318.1) and *E.* *coli* SOD^63^ (E.SOD, GenBank: NP_416173.1) were codon optimized, synthesized as described above and used as positive controls for SOD enzymes. Blank plasmid pET21b was used as a negative control for both MDH and SOD enzymes.

#### Plasmid construction for truncated control sequences

See the Supplementary Methods and Supplementary Table 7.

#### Competent cell preparation and plasmid transformation

Competent cells of *E.* *coli* BL21(DE3) were prepared using the calcium chloride method^88^.

See the Supplementary Methods for details.

#### Protein expression and purification

Protein expression was achieved by diluting the overnight cultures 1:30 into 2.5 ml autoinduction Terrific Broth (TB) medium including trace elements (cat. no. AIMTB0210, Formedium) and supplemented with 100 µg ml^−1^ ampicillin in a 24-well format. All cells were cultivated in 24-well plates in an Eppendorf ThermoMixer C. For MDH expression, cells were grown for 4 h at 37 °C, followed by overnight growth at 16 °C while shaking at 200 rpm. For SOD expression, cells were grown for 4 h at 37 °C, followed by another 3 h at 25 °C with shaking at 200 rpm.

Cells were collected by centrifugation at 3,000*g* for 10 min. Cell pellets were suspended in 200 μl BugBuster reagent (cat. no. 70584, Merck) supplemented with 1 μl 2,000 U ml^−1^ DNase I (cat. no. 79254, Qiagen) and incubated at 37 °C with shaking at 200 rpm for 30 min. After incubation, 10-μl mixtures were aliquoted and kept in −20 °C as the total protein (T) sample for gel electrophoresis. The mixture was centrifuged at maximum speed for 10 min and the pellets were discarded. Then, 10 μl of the supernatant was aliquoted and kept at −20 °C as the soluble protein (S) sample for gel electrophoresis. The supernatants were used for protein purification using the following procedures.

Talon resins (cat. no. 635653, Takara Bio) were washed twice with a binding buffer (50 mM NaH_2_PO_4_, 300 mM NaCl, 10 mM imidazole, pH 7.4) and then suspended in the same volume of binding buffer as the resin bed amount. Talon resin (50 µl) was loaded into Pierce microspin columns (cat. no. 89879, ThermoFisher). Each supernatant sample was added to the loaded column and incubated at 4 °C for 30 min in a thermomixer.

The columns were then centrifuged at 20*g* for 30 s and the flow waste was discarded. Resins were washed with 600 µl of wash buffer three times (50 mM NaH_2_PO_4_, 300 mM NaCl, 20 mM imidazole, pH 7.4) and centrifuged at 20*g* for 30 s each time. Finally, the resins were incubated with 100 µl of elution buffer at 4 °C for 30 min in a thermomixer and proteins were then eluted with centrifugation at 20*g* for 1 min. Another 100 µl of elution buffer was added to repeat the elution steps, and the two portions of elutions were individually mixed. The two eluate fractions were then combined and transferred to a 96-well desalting plate (cat. no. 89807, Thermo Scientific), which was pre-equilibrated with the sample buffer (50 mM NaH_2_PO_4_, 300 mM NaCl, pH 7.4). Protein samples were kept at −80 °C after adding 1⨯ protein-stabilizing cocktail (cat. no. 89806, Thermo Scientific). Then, 10 μl of the proteins was aliquoted and kept at −20 °C as the purified protein (P) sample for gel electrophoresis.

For enzymes from round 2 and round 3 and the truncated enzymes from the round 2 pretest, protein concentrations were measured by Qubit Protein Assay (cat. no. Q33211, Thermo Scientific).

#### Gel electrophoresis

Total, soluble and purified proteins of each sample were mixed with 1⨯ loading buffer (4⨯ loading buffer recipe: 0.2 M Tris-HCl, 0.4 M DTT, 277 mM SDS, 6 mM bromophenol blue, 4.3 M glycerol) and then heated at 85 °C for 5 min in a PCR cycler. Denatured proteins were analyzed by SDS–PAGE with precast gels (cat. no. WG1403A, Thermo Scientific), followed by Coomassie staining with InstantBlue (cat. no. ISB1L-53, Kem-en-tec). Spectra multicolor broad-range protein ladder (cat. no. 26634, Thermo Scientific) was also loaded to analyze the protein sizes.

#### Enzymatic assay

To test for MDH activity, 2 μl or 100 μg ml^−1^ of purified protein in round 1 was added to a reaction mixture containing approximately 1.5 mM NADH (cat. no. 10128023001, Merck), 2.0 mM oxaloacetic acid (cat. no. O4126, Sigma) and 20 mM HEPES buffer (pH 7.4). Assays were performed in triplicate in a 96-well format. All components were added using multichannel pipettes to avoid the reaction time lag of each well. The final reaction volume was 100 µl, and the reaction was carried out at room temperature in a transparent 96-well microplate (cat. no. 0020821, Sarstedt). MDH activity was measured in triplicate by following NADH oxidation to NAD^+^, with an absorbance reading at 340 nm performed in kinetics mode for 15 min in a BMG Labtech SPECTROstar nano spectrophotometer. Unspecific oxidation of NADH was monitored in the no-substrate controls, and these values were subtracted from the other samples. Conversion from absorption values to NADH concentration was carried out using Beer−Lambert law *c* = *A*/(*d* ⨯ *ε*), in which the extinction coefficient *ε* value is 6.22 mM^−1^ cm^−1^, and the path length for 100 μl in a 96-well plate (*d*) is 0.29 cm. For samples that did not show any catalytic activities, a tenfold volume, which is 20 μl of purified proteins, was used to perform the assay for a second time.

For MDH in round 2 and round 3, 20 μg ml^−1^ enzymes together with the positive-control MDH4 were used in the assay as described above for quantitative comparison of catalytic activities, excerpt for samples 1564 and 1546 from round 2, for which the concentration of 0.2 μg ml^−1^ was used due to low protein yields.

SOD activity was measured with a SOD assay kit (cat. no. 19160, Sigma) in a 96-well format, and all components were added using multichannel pipettes to avoid the reaction time lag of each well. For SOD from round 1, an aliquot (2 µl) of purified protein was added to each well containing 98 µl working solution. Assays of each sample were performed in triplicate and in one ‘No XO’ well. xanthine oxidase working solution (10 μl) was added to each well at the end, except for the ‘No XO’ wells. ‘No SOD’ and ‘blank’ assays were also performed in triplicate. ‘No SOD’ wells contained 10 µl dilution buffer, 80 µl working solution and 10 μl xanthine oxidase working solution, while ‘blank’ wells contained 20 µl dilution buffer and 80 µl working solution. Plates were incubated in the plate reader, which was preset at 37 °C. Absorbance at 450 nm was measured in the kinetics mode for 30 min. For proteins that did not show any catalytic activity, a tenfold volume of 20 μl of purified proteins was used to perform the assay a second time.

For SOD from round 2 and round 3, 5 μg ml^−1^ of enzymes were used in the assay as described above for quantitative comparison of catalytic activity.

To assay the truncated proteins, 85 μg ml^−1^ of all samples were used in the enzymatic assay.

For details on the lysozyme assays see the Supplementary Methods.

### Data analysis

For MDH, the absorbance value was plotted over time. The absorbance values of all samples at the endpoint of the assay were compared to the negative control by *t*-test analysis. Samples were considered active if the end absorbance value was significantly lower than that of the negative control, *P* ≤ 0.05.

For SOD, enzyme activity was measured as the percentage inhibition of the rate of WST-1 formazan formation and calculated using the following equation with absorbance value at 20 min. The inhibition rate was compared to the negative control by the *t*-test, and those with activity significantly higher than the negative control were considered active with *P* ≤ 0.05.

SOD activity (inhibition rate %) = ((*A* − *B*) − (*C* − *D*))/(*A* − *B*) ⨯ 100, where *A* is the absorbance value of the ‘no SOD’ control, *B* is the absorbance value of the blank, *C* is the absorbance value of the sample and *D* is the absorbance value of the ‘no XO’.

Assay data were analyzed using GraphPad Prism v8.0.0 for Windows, GraphPad Software (www.graphpad.com).

#### Semiquantitative comparisons of enzyme activities

Data from round 3 enzyme assays using 20 µg ml^−1^ MDH or 5 µg ml^−1^ SOD, as described above, were used for semiquantitative comparisons of enzyme-specific activity (Fig. 3d).

For MDH, MDH4 was used as a wild-type positive control, and for SOD, hSOD, paSOD and E.SOD were used as wild-type positive controls.

For MDH, absorbance at 340 nm was converted to NADH concentration and the average difference in the concentration between the 0 and 90 s time points of the assay was used as a measure of enzyme activity. Some enzymes, including the MDH4 control, converted substrate very quickly, such that most of the substrate was converted before the first time point. Therefore, we replaced any values below 275 µM at time 0 with the mean value from the negative control. Values were averaged over three technical replicates and divided by the average of the MDH4 samples.

For SOD, the inhibition rate (%), calculated as described above, was used as a measure of enzyme activity. Values were averaged over three technical replicates and divided by the average of the hSOD, paSOD and E.SOD samples.

### Reporting summary

Further information on research design is available in the Nature Portfolio Reporting Summary linked to this article.

## Online content

Any methods, additional references, Nature Portfolio reporting summaries, source data, extended data, supplementary information, acknowledgements, peer review information; details of author contributions and competing interests; and statements of data and code availability are available at 10.1038/s41587-024-02214-2.

## Supplementary information

Supplementary Information Supplementary Methods, Figs. 1–35 and Tables 1–5 and 7.

Reporting Summary

Supplementary Table 6 Results of lysozyme expression experiments.

## Figures and Tables

**Extended Data Table 1:**
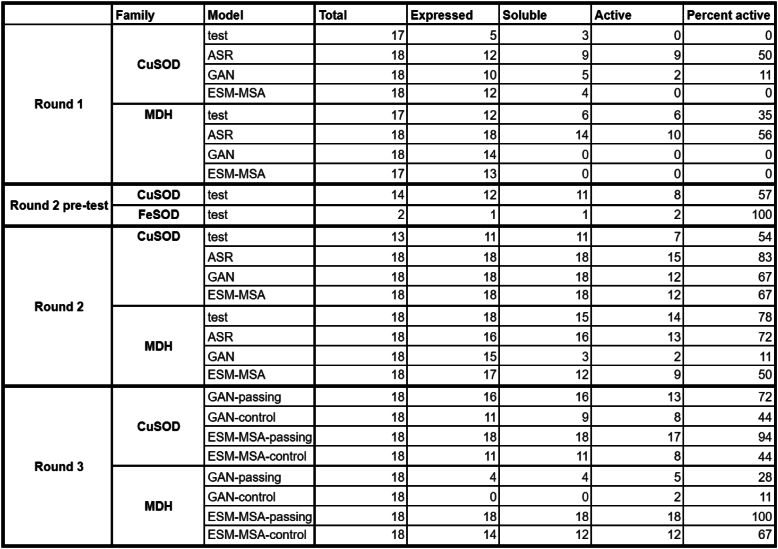
CuSOD and MDH experimental results from all rounds

Expressed: new visible band on SDS-PAGE gel of total protein compared with empty vector control. Soluble: new visible band on SDS-PAGE gel of soluble protein compared with empty vector control. Active: Measured activity significantly different from empty vector control. In some cases, no bands were visible, but activity was detected. For some rows, the total is less than 18, for the following reasons: in Round 1, three of the genes could not be synthesized by Twist; in Round 2, we selected only 13 CuSOD test sequences because we had already tested some similar natural sequences in Round 2 pre-test.

## Data Availability

Training sequences were curated from sequences available from UniProt (release 2022_05) (https://ftp.uniprot.org/pub/databases/uniprot/previous_releases/release-2022_05/knowledgebase/), the NCBI Transcriptome Shotgun Assembly database (https://www.ncbi.nlm.nih.gov/genbank/tsa/) or Mgnify (2022_05) (https://ftp.ebi.ac.uk/pub/databases/metagenomics/peptide_database/2022_05/). All generated sequences, curated natural sequences, train/test splits, predicted structures, metrics scores, phylogenetic trees and tabulations of experimental results are available on Zenodo (10.5281/zenodo.7688667)^89^.

## Code availability

Code for regenerating figures and links to Colab notebooks for calculating metrics and generating sequences using ESM-MSA are available on Github (https://github.com/seanrjohnson/protein_scoring)^90^. Locally executable code for generating sequences from ESM-MSA is also available on GitHub (https://github.com/seanrjohnson/protein_gibbs_sampler)^91^.
