# Author Correction: Computational scoring and experimental evaluation of enzymes generated by neural networks

**DOI:** 10.1038/s41587-024-02275-3

**Published:** 2024-05-20

**Authors:** Sean R. Johnson, Xiaozhi Fu, Sandra Viknander, Clara Goldin, Sarah Monaco, Aleksej Zelezniak, Kevin K. Yang

**Affiliations:** 1 New England Biolabs, Ipswich, MA USA; 2 Department of Life Sciences, Chalmers University of Technology, Gothenburg, Sweden; 3 Institute of Biotechnology, Life Sciences Centre, Vilnius University, Vilnius, Lithuania; 4 Randall Centre for Cell & Molecular Biophysics, King’s College London, Guy’s Campus, London, UK; 5 Microsoft Research, Cambridge, MA USA

**Keywords:** Machine learning, Protein design, Protein sequence analyses

Correction to: *Nature Biotechnology* 10.1038/s41587-024-02214-2, published online 23 April 2024

In the version of the article initially published, Sarah Monaco’s affiliation was incorrect and has now been amended to “Unaffiliated”. Additionally, the text “Sarah Monaco is currently an employee and shareholder of Invitae” has been added to the Competing interests section. These changes have been made to the HTML and PDF versions of the article.

