# Supplementary material for: Computational scoring and experimental evaluation of enzymes generated by neural networks
Source: Nat Biotechnol. 2024 Apr 23;43(3):396–405. doi: 10.1038/s41587-024-02214-2 (PMC11919684; doi:10.1038/s41587-024-02214-2)
Supplement: Supplementary file 2 — Reporting Summary [file 41587_2024_2214_MOESM2_ESM.pdf]

nature portfolio

Corresponding author(s): Aleksej Zelezniak, Kevin K Yang

Last updated by author(s): Mar 12, 2024

Reporting Summary

Nature Portfolio wishes to improve the reproducibility of the work that we publish. This form provides structure for consistency and transparency in reporting. For further information on Nature Portfolio policies, see our Editorial Policies and the Editorial Policy Checklist.

Statistics

For all statistical analyses, confirm that the following items are present in the figure legend, table legend, main text, or Methods section.

n/a	Confirmed	
☐	☒	The exact sample size (*n*) for each experimental group/condition, given as a discrete number and unit of measurement
☐	☒	A statement on whether measurements were taken from distinct samples or whether the same sample was measured repeatedly
☐	☒	The statistical test(s) used AND whether they are one- or two-sided<br>*Only common tests should be described solely by name; describe more complex techniques in the Methods section.*
☒	☐	A description of all covariates tested
☒	☐	A description of any assumptions or corrections, such as tests of normality and adjustment for multiple comparisons
☐	☒	A full description of the statistical parameters including central tendency (e.g. means) or other basic estimates (e.g. regression coefficient) AND variation (e.g. standard deviation) or associated estimates of uncertainty (e.g. confidence intervals)
☐	☒	For null hypothesis testing, the test statistic (e.g. *F*, *t*, *r*) with confidence intervals, effect sizes, degrees of freedom and *P* value noted<br>*Give P values as exact values whenever suitable.*
☒	☐	For Bayesian analysis, information on the choice of priors and Markov chain Monte Carlo settings
☒	☐	For hierarchical and complex designs, identification of the appropriate level for tests and full reporting of outcomes
☐	☒	Estimates of effect sizes (e.g. Cohen's *d*, Pearson's *r*), indicating how they were calculated

Our web collection on statistics for biologists contains articles on many of the points above.

Software and code

Policy information about availability of computer code

Data collection	Sequences used in the analyses were obtained from UniProt (release 2022_05), NCBI Transcriptome Shotgun Assembly database, or Mgnify (2022_05).
Data analysis	Assay data was analyzed using GraphPad Prism 8.0.0<br>Additional custom python scripts for data analysis are available at: https://github.com/seanrjohnson/protein_gibbs_sampler<br>https://github.com/seanrjohnson/protein_scoring

For manuscripts utilizing custom algorithms or software that are central to the research but not yet described in published literature, software must be made available to editors and reviewers. We strongly encourage code deposition in a community repository (e.g. GitHub). See the Nature Portfolio guidelines for submitting code & software for further information.

Data

Policy information about availability of data

All manuscripts must include a data availability statement. This statement should provide the following information, where applicable:

- Accession codes, unique identifiers, or web links for publicly available datasets
- A description of any restrictions on data availability
- For clinical datasets or third party data, please ensure that the statement adheres to our policy

Training sequences were curated from sequences available from UniProt (release 2022_05) (https://ftp.uniprot.org/pub/databases/uniprot/previous_releases/

release-2022_05/knowledgebase/),NCBI Transcriptome Shotgun Assembly database (https://www.ncbi.nlm.nih.gov/genbank/tsa/), or Mgnify (2022_05) (https://ftp.ebi.ac.uk/pub/databases/metagenomics/peptide_database/2022_05/).
All generated sequences, curated natural sequences, train/test splits, predicted structures, metrics scores, phylogenetic trees, and tabulations of experimental results are available at a Zenodo deposit (https://doi.org/10.5281/zenodo.7688667)

Human research participants

Policy information about studies involving human research participants and Sex and Gender in Research.

Reporting on sex and gender	not relevant
Population characteristics	not relevant
Recruitment	not relevant
Ethics oversight	not relevant

Note that full information on the approval of the study protocol must also be provided in the manuscript.

Field-specific reporting

Please select the one below that is the best fit for your research. If you are not sure, read the appropriate sections before making your selection.

☒ Life sciences ☐ Behavioural & social sciences ☐ Ecological, evolutionary & environmental sciences

For a reference copy of the document with all sections, see nature.com/documents/nr-reporting-summary-flat.pdf

Life sciences study design

All studies must disclose on these points even when the disclosure is negative.

Sample size	All samples were processed in one batch for protein expression and purification. For enzymatic assays, the sample size was chosen considering practicalities when measuring assays in 96-well plates.
Data exclusions	All data points are included in the study.
Replication	All measurements were done using at least three biological replicates. Biological replicates were performed for each sample for protein expression and purification twice. Successfully purified proteins were continued for the following SDS gel and enzymatic assays. All enzymatic assays were performed with technical triplicates for each sample. All attempts at replication were successful.
Randomization	Not relevant, all measurements within each round were done on the same plate.
Blinding	We did not performed blinding as all samples were treated within the same protocol in 96 well plates without group bias.<br>Experiments of expression, purification and enzymatic assay were blinded until data analysis.

Reporting for specific materials, systems and methods

We require information from authors about some types of materials, experimental systems and methods used in many studies. Here, indicate whether each material, system or method listed is relevant to your study. If you are not sure if a list item applies to your research, read the appropriate section before selecting a response.

Materials & experimental systems

n/a	Involved in the study	
☒	☐	Antibodies
☒	☐	Eukaryotic cell lines
☒	☐	Palaeontology and archaeology
☒	☐	Animals and other organisms
☒	☐	Clinical data
☒	☐	Dual use research of concern

Methods

n/a	Involved in the study	
☒	☐	ChIP-seq
☒	☐	Flow cytometry
☒	☐	MRI-based neuroimaging
